# A grounded theory exploration of the enablers and barriers of public healthcare access for people with comorbid serious mental and chronic physical illnesses in Jamaica

**DOI:** 10.1371/journal.pone.0309678

**Published:** 2024-08-30

**Authors:** Patrice Whitehorne-Smith, Kunal Lalwani, Robyn Martin, Gabrielle Mitchell, Ben Milbourn, Wendel Abel, Sharyn Burns

**Affiliations:** 1 School of Population Health, Curtin University, Bentley, Western Australia, Australia; 2 School of Global, Urban, and Social Studies, RMIT University, Melbourne, Australia; 3 Department of Community Health and Psychiatry, University of the West Indies, Kingston, Jamaica; 4 School of Public Health, University of York, Leeds, United Kingdom; 5 School of Allied Health, Curtin University, Bentley, Western Australia, Australia; Hamadan University of Medical Sciences School of Dentistry, ISLAMIC REPUBLIC OF IRAN

## Abstract

Chronic physical illnesses (CPI) are highly prevalent among people with serious mental illnesses (PWSMI) yet people in this population experience significant challenges accessing healthcare. This study utilised a constructivist grounded theory approach to collect and analyse data related to the enablers and barriers to public healthcare access for PWSMI & CPI. Data were collected through semi-structured interviews conducted with fifty-seven participants comprising PWSMI &CPI and their caregivers, health policymakers, primary care physicians, psychiatrists, and mental health nurses. Enablers and barriers to healthcare access were represented using a socio-ecological model consisting of five levels: wider society, health system, clinician, family and community, and individual. Jamaica’s free public healthcare system was the most pronounced enabler of healthcare access, while poverty, stigma, and discrimination were the most pronounced barriers. Factors such as social support, time, clinician beliefs, attitudes and training, and individual characteristics were identified as consisting of dimensions that were both enablers and barriers to healthcare access. These findings indicated that factors that influenced healthcare access for PWSMI & CPI were aligned with the social determinants of health. Improved healthcare access for PWSMI & CPI necessitates strategies that incorporate a multi-sectoral approach to address social and environmental factors influencing healthcare access across all levels of the socio-ecological model.

## Introduction

A growing body of evidence indicates that people with serious mental illness (PWSMI) such as schizophrenia and other psychotic disorders, bipolar disorder, and moderate to severe depressive disorders are at increased risk of developing chronic physical illnesses (CPI) [1–3]. The CPI most prevalent among PWSMI include cardiovascular diseases, diabetes mellitus, and cancers [4–7]. Similarly, individuals with CPI experience increased suicidal thoughts, anxiety, and depressive symptoms in comparison to the general population [4, 8]. The co-occurrence of serious mental illness (SMI) and CPI has been linked to their shared aetiology often resulting from the effects of psychotropic medications as well as the impact of social determinants of health [5, 9–13].

Social determinants of health refer to the circumstances in which people are born, grow, work, live, and age; and the economic, social, environmental, and political forces and systems that define and shape their experiences of daily life [14, 15]. Healthcare access has been identified as an important social determinant of health, with reduced access to healthcare resulting in poorer health outcomes, especially among PWSMI [16–18]. Moreover, healthcare access is also affected by other social determinants such as poverty, housing instability, and limited access to transportation, which play a significant role in the health inequalities experienced by PWSMI [8, 19].

In this study, healthcare access represents a multidimensional construct that involves both features of the health service as well as individual characteristics that affect people’s ability to engage and interact with health services [20, 21]. Levesque et al. [20] identified five interrelated dimensions of health service accessibility, (1) approachability (services are identifiable), (2) acceptability (cultural appropriateness of utilising services), (3) availability and accommodation (services are reachable and able to facilitate the delivery of care), (4) affordability (whether costs are subsidised), and (5) appropriateness of care (adequacy and quality of the health service). The five dimensions of health service accessibility interact with five parallel individual abilities that help to determine whether a person’s health needs are met, this consists of the individual ability; (1) to perceive (able to recognise health needs), (2) seek (capacity to choose to seek care based on culture and values), (3) reach (access to a means of transportation), (4) pay (able to afford health service), and, (5) obtain care (able to participate and engage in health decision-making) for their health need [20].

Numerous social factors have been identified as enabling or restricting healthcare access for PWSMI [22]. For example, healthcare access for PWSMI is improved in countries that have clear mental health policies, legislation, strategic plans, and programs [22, 23]. Integrated mental healthcare models, especially those that are community-based, have also demonstrated increased healthcare access for PWSMI & CPI [19, 24, 25]. When clinicians (doctors and nurses) demonstrate sound knowledge, positive attitudes, and practices towards PWSMI & CPI and when these individuals have familial and social support, they report better healthcare access [26–28]. Individual characteristics such as being female, possessing good health literacy, previously accessing mental health services, and having good experience with healthcare providers also enable healthcare access among this population [29–32].

On the other hand, stigma and discrimination are recognised as the greatest barrier to healthcare access for PWSMI globally [33, 34]. Stigma is a social process that attributes negative characteristics towards a specific group based on prejudice and is often manifested as discrimination [35, 36]. Mental health stigma has been reported to contribute to the lack of political will and priorisation of financial investment in mental health services and to legislative frameworks that foster discrimination and social exclusion of people with mental illnesses [37, 38]. Limited social support, social isolation, exclusion and marginalisation of PWSMI are all associated with mental health stigma [39, 40]. These experiences contribute to the self-stigma experienced by many people living with mental illness resulting in shame, masking, and/or poor help-seeking among this population [39, 40]. Issues of stigma and discrimination related to mental health are especially prominent in low-and-middle-income countries with limited financial and human resources invested in mental health, resulting in wide treatment gaps and insufficient healthcare access for PWSMI [38]. In addition, other social determinants of health such as poverty and poor health literacy common among PWSMI create barriers to help-seeking and subsequent healthcare access [41–43].

Jamaica represents an upper-middle-income-country that has taken intentional steps through the implementation of community-based mental health care that is incorporated into primary care to improve healthcare access for PWSMI despite limited financial and human resources [44, 45]. Jamaica also allocates a little over 5% of the health budget to mental health care, which is higher than the median reported expenditure of high and low-income countries on mental health services [46]. Other measures implemented in Jamaica include the provision of specialised mental health services in all hospitals and most community health centres; free public healthcare for doctor consultations (including specialists); the inclusion of subsidised essential pharmacological medication; and redesigning the medical school curriculum to improve mental health competence [45–47].

Notwithstanding these improvements, the treatment and healthcare access gap for PWSMI continues to be wide in Jamaica [46]. Research conducted in Jamaica suggests this can be attributed to limitations in financial and human resources; effects of stigma and discrimination; clinicians’ attitudes towards mental health; as well as factors such as poverty; and low health and mental health literacy [45, 46, 48–52]. However, there is an absence of research that directly explores the enablers and barriers to healthcare access for PWSMI & CPI. This is a significant research gap considering the outcomes associated with these co-morbidities. Furthermore, there is a paucity of mental health research in Jamaica that includes the perspectives of PWSMI about their healthcare experience. The World Health Organisation [19, 53] has called for the incorporation of the voices of people with lived experience of mental illness, and various relevant stakeholder in national mental health research. Research of this nature facilitates comprehensive exploration of mental health issues that is situated in the local context, and allows for the tailoring of intervention based on the specific needs and context of that country [19]. Consequently, the current study sought to identify and describe the enablers and barriers to healthcare access for PWSMI & CPI from the perspective of several stakeholder groups.

## Materials & methods

### Study design

A qualitative constructivist grounded theory approach guided this study [54, 55]. To the best of the authors knowledge this study was the first to explore the enablers and barriers to healthcare access for PWSMI & CPI in Jamaica, as such a qualitative approach enabled a deep and rich exploration of the topic [56]. Charmaz [54, 55] constructivist grounded theory approach allowed for a careful, detailed and systematic process of exploration of psychological and social processes within the context of the healthcare access. Additionally, the socio-ecological model (SEM) was used as a conceptual framework to explore the Jamaican public health sector as a system consisting of multilevel factors that affect healthcare access for PWSMI &CPI [57]. The conceptual framework of the SEM guided the identification of relevant stakeholder groups and provided context to the data analysis process and framing of the study findings. The involvement of multiple stakeholder groups in the study added a level of complexity to the data analysis process that could be addressed using this methodology and led to the development of a conceptual model grounded in the study findings [58].

### Setting

Jamaica is an English-speaking Caribbean island nation currently classified as an upper-middle-income country with a population of 2.82 million [59]. The most recent available estimates identify schizophrenia and bipolar disorder as the most prevalent serious mental illness in the population, affecting 1.4% and 1.3% of the population respectively [60]. Most PWSMI attend public community health centres to access health services [45]. There are 315 community health centres located island-wide offering a range of health services through curative and specialty clinics including mental health clinics [61]. Psychiatrists and/or mental health nurses manage mental health clinics in primary care with the support of psychiatric aides who form the core of the mental health team; a few teams include a psychologist and/or social worker [45]. Mental health teams engage in the screening and management of mental illnesses with a focus on SMI, crisis management, home visits, and health promotion activities [62]. Secondary and tertiary public healthcare is offered through 23 hospitals across the island [61]. Within the Jamaican public health system, service users are referred to as patients; therefore the language of this paper acknowledges this local context.

### Participants

This study comprised six participant groups. The first two groups consisted of the health service users of interest, namely; (1) PWSMI & CPI and (2) caregivers of PWSMI & CPI. The inclusion criteria for the PWSMI& CPI were, a pre-existing SMI diagnosis made by a psychiatrist, no reported psychiatric crisis in the past seven days, and, reported at least one CPI. For caregivers of PWSMI & CPI, the inclusion criteria was that they had a family member whom they provide care for with co-morbid SMI and CPI. The remaining four groups consisted of health professionals who had varying levels of involvement in health service delivery to PWSMI & CPI. These groups included, (3) health policymakers (HPMs), (4) primary care physicians (PCPs), (5) psychiatrists (Psychs) and (6) mental health nurses (MHNs). Inclusion criteria for health professional participant groups one to four were: having a current role in the public health system for a minimum of one year, or for Psychs, at least a final year resident in Psychiatry. Participants from all groups were aged 18 years or older. Based on the diverse of the participant groups, this paper uses different terms to categorise participants. The term health professionals apply to participants from groups three to six represent all participants working in the health system. The term clinicians is used to denote participant groups four to six while mental health clinicians refers to participants from groups five and six.

### Recruitment & data collection

Ethical approval for the study was provided through the Ministry of Health and Wellness Jamaica’s Medico-legal Ethics Committee (2019/49), Curtin University Human Research Ethics Committee (HRE 2020–0022), and the University of the West Indies, Faculty of Medical Sciences Ethics Committee (#ECP 101, 19/20). Purposive and snowball sampling techniques were used to recruit participants [56]. Health policymakers were purposively selected based on their involvement with PWSMI & CPI and were recruited via direct email invitation. Psychiatrists and PCPs were recruited through their expression of interest to a flyer circulated via relevant medical associations as well as through snowball sampling. Recruitment of PWSMI & CPI and their cargeivers was achieved through an expression of interest to a flyer circulated in a mental health support group as well as via study sensitisation sessions conducted at eighteen mental health clinics island-wide.

Before taking part in the study all participants who met the inclusion criteria for each group received participant information sheets outlining details of the study and provided written informed consent to be interviewed and audio recorded. For members of the PWSMI & CPI, the research team who were trained Jamaican psychologists asked pre-screening questions to capacity to provide consent by enquirying about their experience of a psychiatric crisis in the past seven days and their current mental health state. This method of informed consent was approved by all three ethical committees. Non-identifying demographic information was collected via a pre-interview checklist. Semi-structured interviews were conducted with the aid of interview guides and allowed for the exploration of enablers and barriers and discussion around unanticipated topics presented by participants [63]. Each interview guide consisted of questions relevant to the participant group and included questions that explored aspects of diagnoses, screening and management, enablers and barriers to healthcare access for PWSMI & CPI as well as perceptions around the health system response to PWSMI & CPI. For example, PWSMI & CPI were asked “what are some circumstances that have allowed you to be able to access the care you needs for chronic physical illness?”. This question directly considered enablers to healthcare access for CPI. They were also asked “what are some circumstances that have made it difficult for you to access the care you needs for chronic physical illness?” as a measure of barriers to healthcare access. All interview guides included a variation of these question. Interviews were conducted by the lead author and lasted between 30–60 minutes. Members of the PWSMI & CPI and caregivers groups received a mobile recharge voucher valued at $500 (USD $3.50) as a token of appreciation for participating in the study. Data was collected from March to November 2020. During this time, the COVID-19 pandemic was ongoing, interviews were conduced via telephone or video conferencing in keeping with physical distancing guidelines [64].

### Data analysis

Audio recordings from interviews were transcribed verbatim and where possible returned to participants with email access for their review. A constructivist grounded theory approach was used to guide the data analysis with emphasis on identifying and understanding emerging enablers and barriers to healthcare access for PWSMI & CPI [55]. Data was coded using NVivo version 14 software [65]. The full transcript for each participant was reviewed multiple times using line-by-line coding to identify initial codes [66]. Codes were then refined into focused codes based on case-by-case comparisons and negative case analysis within and across the six participant groups with the aid of field notes and memos that guided the analytic process [58, 66]. The process indicated a pattern of common focused codes and then categories across participant groups, which reflected a SEM of enablers and barriers across five levels. Theoretical codes were then generated to integrate and synthesise the categories into themes representing a localised grounded theory of enablers and barriers to healthcare access for PWSMI & CPI [54, 55].

To ensure the rigour of the study and data analysis process several measures were taken. Detailed records of the study protocol, data collection and analysis were maintained through the use of field notes and memos which safeguarded the dependability of study [58]. All transcripts were transcribed and where possible shared with participants for their review and feedback. There was also prolonged engagement with the data, with data triangulated within and across participant groups [56]. All codes and categories were assigned by PWS and independently reviewed by SB and BM. The research team engaged in bracketing and frequent reflexive discussions about emergent findings to ensure that interpretations were rooted in the data and to enhance credibility and confirmability [66]. Participants were purposively sampled and the number of participants sampled and the amount of data gathered were deemed sufficient to meet the research objectives as codes became repetitive in later interviews ensuring the transferability of the results [67, 68]. As an added measure to secure the overall rigour of the study findings, a six-member stakeholder reference group, consisting of health and mental health researchers and clinicians as well as a PWSMI and a caregiver provided oversight of the study [69]. The stakeholder reference group engaged in meetings and discussions throughout the data collection process, provided face validity for the study instruments and made recommendations related to the study data collection process, presentation of study findings, and the study dissemination plan.

## Results

Thirteen males and 44 females (N = 57) participated in the study (Table 1). The most common age groups were 51–60 years (n = 18, 31.5%), followed by 31–40 years (n = 14, 24.6%). Among health professionals, most participants had worked in their current role for 6–10 years (n = 11, 37.9%) or 1–5 years (n = 9, 31%). Amongst participants in the PWSMI & CPI and caregiver groups, primary and secondary level education was most commonly reported (n = 18, 64.2%). The most common SMI diagnoses reported in these groups were schizophrenia (n = 16, 57%), followed by ‘unknown’ representing those who did not know their own or their relative’s diagnosis (n = 6, 21.4%). The most frequently reported CPI was hypertension (n = 10, 35.7%), followed by the combination of hypertension and diabetes (n = 7, 25%) and other CPIs (n = 7, 25%).

**Table 1:** Participant characteristics (N = 57).

Characteristic	Health policymakers (n = 4)	Primary care physician (n = 9)	Psychiatrists (n = 11)	Mental health nurses (n = 5)	PWSMI & CPI (n = 23)	Caregivers (n = 5)	Total (N = 57) %	
*Gender*								
male	2	3	2	1	5		13	22.8%
female	2	7	9	4	18	5	45	78.9%
*Age group*								
25–30 years		3			1		4	7.0%
31–40 years		4	9		1		14	24.6%
41–50 years	1	2	1	3	6		13	22.8%
51–60 years	2		1	2	12	1	18	31.6%
Over 60 years	1				3	4	8	14.0%
*Education level*								
Primary					6	1	7	12.3%
Secondary					9	2	11	19.3%
Vocational					5		5	8.8%
Tertiary	4	9	11	5	3	2	34	59.6%
*Number of years working in role* *								
1–5 years	2	6		1			9	15.8%
6–10 years	1	1	9				11	19.3%
10–20 years		1	2	1			4	7.0%
over 20 years	1	1		3			5	8.8%
SMI Diagnosis** (*personal diagnosis or that of relative)*								
Schizophrenia					14	2	16	28.1%
Schizoaffective disorder					1		1	1.8%
Bipolar disorder					1		1	1.8%
Major Depression					1	1	2	3.5%
Schizophrenia & Bipolar disorder					1		1	1.8%
Drug induced psychosis						1	1	1.8%
Unknown					5	1	6	10.5%
CPI Diagnosis** *(personal diagnosis or that of relative)*								
Hypertension only					9	1	10	17.5%
Diabetes only					3	1	4	7.0%
Hypertension & Diabetes					7		7	12.3%
Other CPIs					4	3	7	12.3%

* characteristics applicable only workers in the public health system

** characteristics applicable only to PWSMI & CPI and caregivers

**Fig 1:**
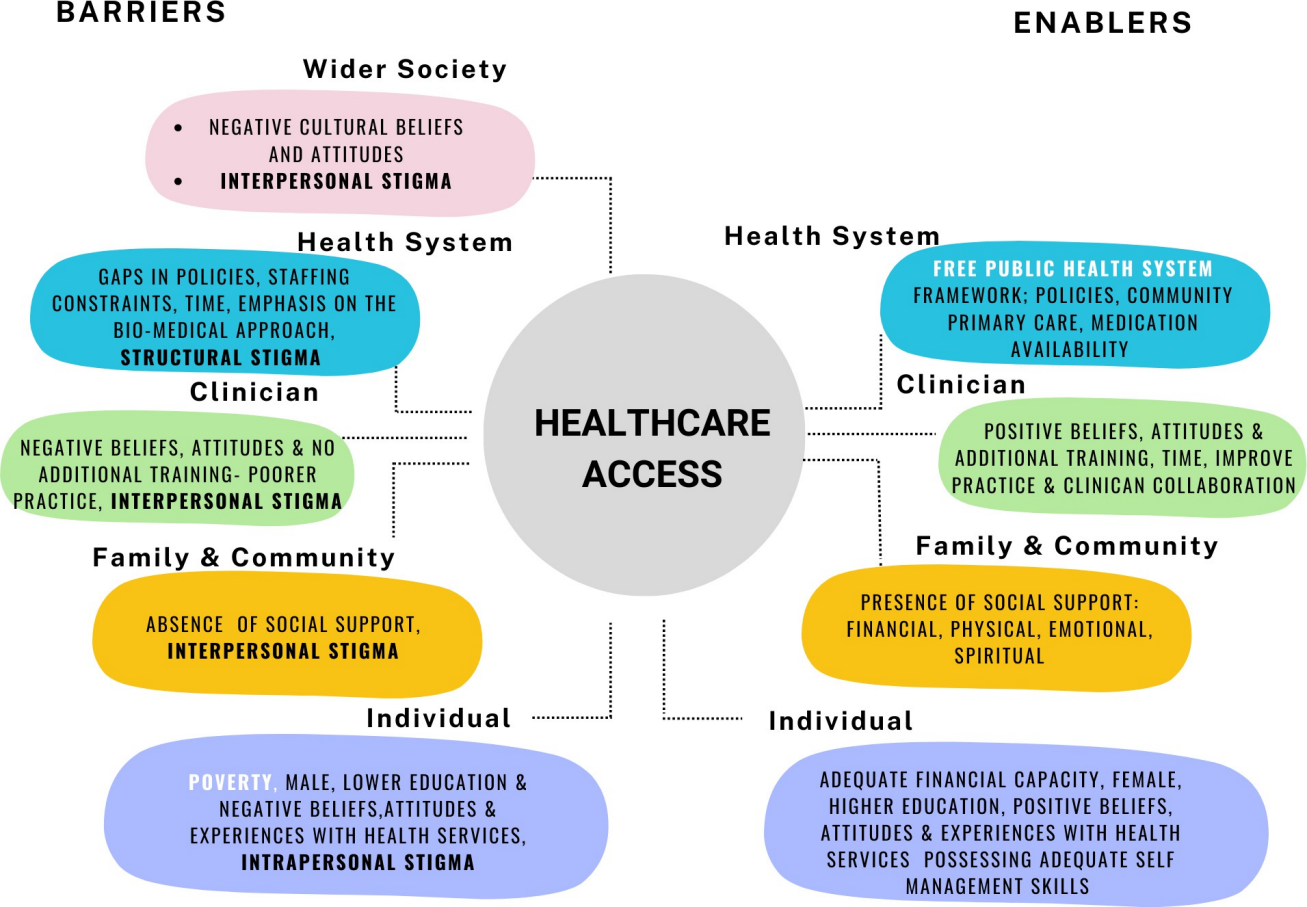
Socio-ecological model of enablers and barriers to public healthcare access for PWSMI & CPI. (Note: Text in white are the primary enablers and barriers).

The enablers and barriers to public healthcare access for PWSMI & CPI identified an interplay of themes spanning across five categories representing five levels of a SEM (Fig 1). These levels included: (1) Wider society—representative of cultural views and attitudes, (2) health system—representative of the health system structure and operation, (3) clinicians—representative of clinicians’ beliefs attitudes, training, and practices, (4) family and community—representative of social support available to PWSMI, (5) individual—representative of individual characteristics that influence healthcare access. Themes and sub-themes are presented according to the levels they operate at in the SEM. All themes were presented as both enablers, and barriers except, two: cultural attitudes, and stigma and discrimination, which are solely barriers. Table 2 illustrates how various enablers and barriers affect the dimensions of healthcare accessibility at each level of the SEM.

**Table 2:** Socio-ecological model of enablers and barriers to healthcare access for PWSMI & CPI.

Level of socio-ecological system	Categories	Enablers	Barriers	Dimension of Access Affected
Across all levels	Stigma and discrimination		Interpersonal stigma	*Health service dimensions*: Acceptability, Appropriateness *Individual abilities*: ability to seek, reach, pay and engage
			Structural stigma	*Health service dimensions*: Acceptability, Appropriateness *Individual abilities*: ability to engage
			Intrapersonal stigma	*Individual abilities*: ability to perceive, seek, reach and engage
Wider society	Negative cultural beliefs and attitudes		Cultural beliefs and attitudes about substance use, causes of illness, obesity and PWSMI	*Health service dimensions*: Acceptability, Appropriateness *Individual abilities*: Ability to perceive, seek, reach and engage
Health system	Free public healthcare	policy and legislation for free healthcare and mental health	Gaps in policies and guidelines related to the screening and management for PWSMI & CPI	*Health service dimensions*: Approachability, availability and accommodation, affordability, appropriateness
		Free physical and mental health consultations	Staffing constraints	*Health service dimension*: Appropriateness *Individual abilities*: Ability to seek, reach, pay and engage
		Co-location of primary healthcare in community settings		*Health service dimensions*: Approachability, acceptability, availability and accommodation, affordability, appropriateness *Individual abilities*: Ability to seek, reach, pay and engage
Level of socio-ecological system	Categories	Enablers	Barriers	Dimension of Access Affected
Health system *(continued)*		Community outreach by the mental health team	Emphasis on the bio-medical approach	*Health service dimension*s: Availability and accommodation, appropriateness *Individual abilities*: Ability to perceive, seek, reach, and engage
		Presence of multidisciplinary teams in some settings		*Health service dimensions*: acceptability, availability and accommodation, appropriateness *Individual abilities*: Ability to perceive, seek, reach, and engage
		Availability of medication		*Health service dimensions*: Affordability, appropriateness of care *Individual abilities*: Ability to pay and engage
Health system & Clinician levels	The impact of time on healthcare access	Clinicians with additional training or experience with mental illness/CPI spend more time	Limited time as a barrier to healthcare access in terms of wait time for patients to be seen and time spent with clinician.	*Health service dimensions*: Availability & accommodation and appropriateness *Individual abilities*: Ability to seek, and engage
Clinician	Clinician beliefs attitudes, training and practices	Positive beliefs and attitudes towards PWSMI & CPI improved practice	Lack of additional training and experience in MI or CPI negatively affected practice	*Health service dimensions*: Acceptability and appropriateness of care *Individual abilities*: Ability to perceive, seek, and engage
		Positive beliefs and attitudes towards PWSMI/CPI improved collaboration between clinical teams	Clinician negative beliefs, attitudes towards SMI or CPI management negatively affected practice	*Health service dimensions*: Acceptability and appropriateness of care Individual abilities: Ability to perceive, seek, and engage
		Positive beliefs and attitudes towards PWSMI & CPI improved patient-centred care and collaboration between clinical teams		*Health service dimension*s: Acceptability and appropriateness of care *Individual abilities*: Ability to perceive, seek, and engage
Level of socio-ecological system	Categories	Enablers	Barriers	Dimension of Access Affected
Family & community	Social support	Presence of social support as an enabler to healthcare access	Absence of social support as a barrier to healthcare access	*Individual abilities*: Ability to perceive, seek, reach, pay and engage
Individual	Financial Capacity	Adequate financial capacity	Poverty	*Individual abilities*: Ability to reach, ability to pay
	Individual beliefs, attitudes	Positive beliefs, attitudes, and experiences with health services	Gender and Education characteristics that bar healthcare access	*Individual abilitie*s: Ability to percieve, ability to seek, ability to engage
	Socio-demographic features	Gender and Education characteristics that enable healthcare access	Negative beliefs, attitudes, and experiences with health services	*Individual abilities*: Ability to perceive, seek and engage
	Health literacy and self-management skills	Self-management skills enable healthcare access		*Individual abilities*: Ability to perceive, seek and engage

### Across all levels of the SEM

Stigma and discrimination were identified as major barriers to healthcare access that existed at all levels of the SEM. Participants descriptions of stigma indicated interpersonal, structural and intrapersonal stigma which resulted in discrimination. The manifestation of stigma and discrimination negatively affected healthcare access on both the health service dimensions and individual abilities dimension (Table 2). On the health services dimensions cultural norms that promoted stigma and discrimination towards PWSMI & CPI reduced the acceptability of utilising health services and the appropriateness of care provided by clinicians. Additionally, for PWSMI & CPI stigma and discrimination negatively affected their ability to perceive, seek, reach and engage with healthcare services due to a fear being labelled by the community or their own family members (Table 3).

**Table 3:** Illustrative quotes of themes and sub-themes represented across the socio-ecological model.

Level of socio-ecological model	Themes	Sub-themes	Number of Participants	Illustrative Quote
Across all levels	***Stigma and Discrimination***		34	So, there’s a lot of negative connotations attached to mental illness, psychiatry, psychology because of how persons might be viewed in the community…, That’s one big thing, Jamaicans are afraid of mental illness and the diagnosis of mental illness… they’re very afraid to let anybody else… whether in their household, or close relatives know about the mental illness because of what it can cause in their work life, or personal life. (PCP #10)
		Interpersonal stigma	30	The relatives don’t like the stigma of their family member being mentally ill, so they might not come with them to the clinic or help them take their medication. Some prefer to know that they are on the streets than at home. (MHN #1)
		Structural Stigma	28	
				***Policy and resource allocation*:** [Psychiatry] is the sort of Black Sheep or forgotten stepsister… it makes it difficult for our clients to access services… you will interact with clinicians in different settings and they will make statements that are obviously indicative of holding a certain level of stigma against you… this [attitude] filters down to all of the various levels of staff. So even when you’re talking about how persons are treated and greeted when they enter an institution by security guards, administrative staff, all of those things can end up impacting people’s well-being and wellness. (Psych #4)
;				***Challenges accessing physical health services***: in text
		Structural Stigma		***Stigma and Discrimination by clinicians and other staff***: I think the stigmatizing that they put on the mentally ill is bad. Especially the nurses who know that mental illness can happen to anyone at any time… They need to talk to the patient in a better way and make us feel more comfortable. They should stop being impatient with us and embarrassing us. They should not want to hurt a person who is mentally ill.” (PWSMI # 19)
		Self-stigma	11	[PWSMI] might not even wait for treatment because they are also in denial. . .they look at it as, I’m mad, as they would call it … acceptance is a big issue…so that leads to no follow-up, which leads to persons not taking medications. (PCP #10)

#### Interpersonal stigma

Participants indicated that societal beliefs and attitudes limited the social support given to PWSMI & CPI and often resulted in their exclusion by family and community members or in PWSMI & CPI withdrawing and becoming socially isolated. Stigma and discrimination were attributed to a general lack of awareness and understanding of mental illness, fear, and a societal belief that PWSMI are ‘aggressive’ and ‘unpredictable’. Several clinicians indicated that these cultural views about mental illness mediated diagnoses of MI because it was seen as an indication that they were “mad,” which was viewed negatively within the community, and created social isolation.

In some communities, people who were labelled as ‘mad’ were ostracised and treated poorly. For some family members the stigma affixed to their relative extended to them and so they withheld their social support as a means of distancing (Table 3). Interpersonal stigma and discrimination towards PWSMI impeded the individuals’ ability to seek, reach, and engage with healthcare. Participants believed that interpersonal stigma led to a delayed presentation to mental health services by PWSMI resulting in symptom exasperation and worsened health outcomes. This form of stigma was also seen as related to structural and intrapersonal stigma and discrimination.

#### Structural stigma

Structural stigma and discrimination towards PWSMI & CPI was identified as a feature of the public health system which manifested in: (1) policy and resource allocation, (2) challenges accessing physical health services, and (3) stigma and discrimination among healthcare clinicians and staff.

*Policy and resource allocation*. Participants believed that structural stigma negatively affected government prioritisation and investment in mental healthcare, including influencing policy frameworks: *“I think stigma is the factor that affects access to care*… *stigma affects the beginning in terms of how we’re approaching the patient*… *including even policymakers*.*” (Psych #5)*. Structural stigma affected PWSMI & CPI, their family members as well as psychiatrists as it influenced the allocation of resources, the placement of psychiatric services in facilities, and even interactions with clinicians from other specialties (Table 3).

*Challenges accessing physical health services*. Hospital settings were noted to be the healthcare context with the highest level of structural stigma. Participants explained that PWSMI & CPI often encountered challenges gaining entry to facilitates, receiving screening and treatment to address their physical health needs, being made to wait longer than other patients for services, and being passed off to mental health services: *“once the patient is mentally ill*, *nothing else counts*. *So even if they go there and say they have whichever other issues*, *the mental illness takes prominence and nobody else remembers what the other issues are that they have*.*”* (MHN #3).

*Stigma and discrimination by clinicians and other staff*. In attempting to access healthcare PWSMI & CPI may experience public ridicule, humiliation, and disrespect which sometimes resulted in them leaving without receiving services and/or not returning for treatment. While issues of stigma and discrimination were more commonly reported by health professionals, a few PWSMI & CPI and family members reported instances of stigma and discrimination by clinicians, health administrative staff, and security personnel (Table 3). In these instances, PWSMI & CPI and their family members attributed the mistreatment to their SMI diagnosis. These participants reported experiences where clinicians disbelieved physical health symptoms, and were denied access to health services.

#### Intrapersonal stigma

Intrapersonal stigma diminished the individual’s ability to perceive, seek, reach, and engage with healthcare. Clinicians indicated that intrapersonal stigma in PWSMI & CPI manifested as ‘denial of their diagnosis’, unwillingness to accept treatment, and/or reluctance to disclose the diagnosis (Table 3). Some PWSMI & CPI explained that they did not share their mental health diagnosis with family members, friends, and even PCPs as they did not want to be viewed differently. These PWSMI & CPI saw mental and physical health as separate issues: *“I don’t want them (PCPs) to be biased and say okay*, *I guess it’s because you’re not well [mentally]*, *why are you asking about your prostate*… *so I treat them separately” (PWSMI #2)*.

### Wider society

At the level of the wider society, some cultural beliefs and attitudes were identified as barriers to healthcare access. The cultural acceptance of the use of alcohol, tobacco, and marijuana as typical aspects of social behaviour even among health professionals was seen as mitigating against the routine screening and management for substance misuse among PWSMI & CPI. This served to reduce PWSMI & CPI’s access to acceptable and appropriate care that addressed substance misuse. Likewise, a few health professionals indicated that healthcare access was affected by cultural beliefs about the origin of SMI and CPI such as “obeah” (witchcraft). Obeah is believed by some to be the origin of physical and mental illnesses:

*“Sometimes they [patients] incorporate it into their psychosis that persons might be “obeahing” [witchcraft] them to make them sick both mentally and physically so they refuse help from doctors*”(*Psych #1)*

These cultural beliefs and attitudes limited individuals’ ability to perceive, seek, and engage with health services as substance misuse was either not recognised or accepted as a health concern, or in the case of obeah not considered to be treatable by health professionals.

### Health system

The free public health system operating at the health system level of the SEM was recognised by the vast majority of participants as the primary enabler of healthcare access for PWSMI & CPI.

The free healthcare system was built on policy and legislation inclusive of physical and mental health care provision, which promoted access across primary, secondary, and tertiary care. Even so, participants were able to identify features of the free public health system that represented enablers as well as barriers to healthcare access for PWSMI & CPI.

#### Enablers in the free public health system

Participants identified four aspects of the free public health system that enabled healthcare access these included: (1) co-location of primary healthcare in community settings; (2) community outreach by the mental health team; (3) availability of free medication, and (4) presence of multidisciplinary teams.

*Co-location of primary and mental healthcare in community*. The co-location of primary care and mental health services within communities island-wide, facilitated the healthcare access on the dimensions of availability and accommodation of health services to PWSMI & CPI. This was especially true for those who lived in rural/remote areas who would otherwise travel great distances to receive health services. It also aided easy referral of patients across clinics and facilitated clinician access to shared patient records (Table 4).

**Table 4:** Illustrative quotes from the SEM level of the health system.

Level of socio-ecological model	Themes	Sub-themes	Number of Participants	Illustrative Quote
Health System	***Enabler is the free public health system***		52	
		Co-location of primary & mental healthcare in community	45	Trying to treat people as close as possible to the home… especially out in the rural parts… the network we have, the way that’s clinics are structured are very effective, added to that, is that we also have a community [mental health] outreach team (MHN #1).
		Community outreach by the mental health team	25	The fact that they send person, nurses, aides, and psychiatrists, doctors into the environment sometimes on these visits to seek out persons who have been seen in the hospital and in the clinics to follow up on them [works well]. (PCP #7).
		Availability of medication	18	In-text
		Presence of multidisciplinary teams	12	The fact that we can have all these other members of the team accessible if I have an issue, I can reach out to them… I have my social work and [if] there’s a social issue with the patient, I can lean on that professional. I have the psychiatrist there… so that works well. (PCP #8).
	Barriers in the free public health system		31	
		Limited time as a barrier to healthcare access	29	***Clinician*:** Most of the time they’re not adhered to [international guidelines] and that’s the honest truth. Because in the clinic setting in the public system, we are limited, in that we don’t have the nursing staff at times to assist with taking the measurements that we may need. Again, the clinic numbers may be large. Patients are outside getting frustrated, so you want to try to move them through as quickly as you can. (Psych #9)
				***Patient*:** It’s just the length of time that I have to wait to see the doctor [that is difficult]. That’s the worst part because… it causes a lot of pain. By the time I see the doctor, I’m in so much pain. I can’t even think straight.” (PWSMI # 1)
		Staffing constraints	26	Well, staffing is an issue… in order to develop good rapport with patients and a good treatment compliance sometimes the patient needs to see the same doctor over again for a good period of time. So, there is an issue with frequent turnover of doctors, and they aren’t staying there long enough and we don’t necessarily have enough staff to do the work as well… as it relates to mental health, we have a paucity of some skills in the system. (HPM # 1)
		Emphasis on the bio-medical approach	18	We give the person medication but they may not have somewhere to live, the patient may not have food to eat and all of those are not addressed necessarily because there’s nobody who is really looking at those issues so the patient goes home diabetic have the right medication but can’t eat the right food, can’t afford the right food to eat so with that diet which is an important part of his management is taken care of similarly with a psychiatric patient. So those support staff mainly psychologists and social workers are really lacking and if you have one of those in the system it helps the overall care of the patient instead of just taking care of them in terms of their medication but also thinking of their psychosocial needs which contributes towards their improvement. (HPM #4)
		Gaps in policies and guidelines	18	sometimes they [patients] can’t even advise you what happened either [at the PCP appointment], they can just say I went to the doctor and they gave me that medication and some may have brought a prescription with them that you need to make out what it is they were possibly diagnosed with given the medications prescribed. (Psych #1)
				Sometimes we find that patients might even have to change medication even though they’re doing well on it because it’s just not available. That in itself, deters the patient, they lack the confidence in this new medication because they were responding so well to one and now they’re not on it anymore. And those are some of the things that can negatively impact the Health Care system as well as the overall patient compliance and positive attitude towards us. (Psych #6)

**Note:** dominant enablers and barriers are written in bold italics

*Community outreach by the mental health team*. Members of the mental health team engage in community outreach activities, respond to crisis calls, and conduct home visits for patients who have not attended their clinic appointments. The mental health teams’ home visits was seen as a vital measure to that enhanced healthcare access on the dimensions of availability and accommodation of health services. Home visits allowed individuals who missed their appointments or who were in crises to access mental health and sometimes, physical health consultation and receive medication in their homes (Table 4).

*Availability of medication*. Medications were often made available free of cost through government-operated pharmacies (DrugServ) frequently co-located at community health centres. This aided PWSMI &CPI and their family members who could not afford medication to gain access to treatment:: *Well*, *I mean the Drug Serv*, *I go through them to get medication*, *so that helps*, *because I can’t afford the tablets other than getting it from the Drug Serv*.*” (PWSMI #21)*.

When medications were unavailable through DrugServ, PWSMI & CPI who had government insurance cards could obtain subsidised medication at a private pharmacies. This facilitated the affordability of healthcare and treatment for PWSMI & CPI.

*Presence of multidisciplinary teams*. Optimal healthcare access occurred in settings where multidisciplinary teams were present, which increased the appropriateness of care provided to patients. These teams included medical staff, dieticians as well as psychologists and/or social workers, or when PCP worked along with the mental health team. However, this was more of an exception than normal practice (Table 4).

#### Barriers to the free public health system

Participants also discussed some barriers to healthcare access for PWSMI & CPI. These included: (1) limited time as a barrier to healthcare access, (2) staffing constraints, (3) gaps in policies and guidelines related to screening and management for PWSMI & CPI and (4) emphasis on the bio-medical approach.

*Limited time as a barrier to healthcare access*. Participants indicated that throughout the public health service, time was an issue for both clinicians and patients due to limited staffing and large patient volumes. Time was related to both the wait time to see patients and time spent in consultation with patients. Health professionals recognised that having limited time with patients resulted in inadequate screening, detection, and management of both SMI and CPIs based on international guidelines. This decreased patients’ access to appropriate care to address their health needs (Table 4).

From the perspective of the PWSMI & CPI and their family members, the wait time to see clinicians was challenging. This was especially true for patients who experienced pain from sitting or standing for long periods as well as the elderly (Table 4). These participant groups described a system that was often involved waiting all-day, with patients arriving early and leaving six to eight hours later. Waiting was not only a problem because of frustration but also because of hunger. Several participants indicated that PWSMI and their family members often did not have money to purchase food while they waited. Some PWSMI & CPI and their family members accepted the system and did not ‘complain’, but a few said that waiting was a deterrent and resulted in them not attending appointments or delaying access to health services.

*Staffing constraints*. Health professionals indicated that limited staff and high staff turnover, especially in primary care was a central barrier to healthcare access for PWSMI & CPI. Participants explained that PWSMI & CPI often did not have a regular doctor as PCPs rotated between health centres. Across participant groups, the cycling of clinicians was noted as affecting the dimension of access related to the appropriateness of care as it presented challenges for rapport building, co-management, and continuity of care (Table 4).

Staffing issues existed across the public health service, particularly with limited specialised staff. Some mental health teams did not have an assigned psychiatrist, especially in rural or remote areas and many did not have a PCP that worked with their team.

*Gaps in policies and guidelines related to the screening and management for PWSMI & CPI*. Although the presence of policies and guidelines related to mental health care enables some access, gaps in these policies and guidelines created barriers to healthcare access, especially related to the appropriateness of care received by PWSMI & CPI. The lack of clear co-management guidelines for SMI & CPI resulted in role ambiguity among PCPs and Psychs regarding the level and type of responsibility for the physical health of these patients. This created inconsistent practices in the screening and management of PWSMI & CPI.

Additionally, a lack of structured engagement and communication protocols between PCPs and mental health teams resulted in clinicians primarily relying on patients to transfer information. Several health professionals indicated that sometimes this method was ineffective, as patients may be unsure of their diagnosis or the name of prescribed medication, resulting in clinicians making assumptions or uninformed decisions when prescribing medications (Table 4).

This was a substantial concern when patients utilised health services other than their community health centres such as hospitals or clinics in other areas. In these circumstances, clinicians were unable to access patient records due to the lack of a centralised electronic database of patient records within the public health system.

*Emphasis on the bio-medical approach*. Participants reflected on the bio-medical approach that focused on the role of doctors and nurses with limited attention to the contribution of allied health professions in improving the healthcare access and outcomes for PWSMI & CPI (Table 4). Additionally, clinicians indicated that over-reliance on medication as the only treatment for PWSMI & CPI coupled with inconsistent medication supply sometimes resulted in sub-optimal treatment. Shortages of medications led to new prescriptions which created challenges for the clinician-patient relationship and patient attitudes towards treatment and compliance (Table 4). These factors reduced the appropriateness of care provided to PWSMI & CPI who also felt there were few alternatives to treatment provided to them outside of medications.

### Clinician

At the clinician level, the training and experience of clinicians influenced their beliefs and attitudes about the management of PWSMI & CPI. This in turn shaped their practices and affected whether their response to patients presented as enablers or barriers to the accessibility of acceptable and appropriate healthcare.

#### Clinician related factors that enable healthcare access

Participants discussed three clinician related factors that were enablers of PWSMI & CPI healthcare access, namely: 1) positive beliefs and attitudes towards PWSMI & CPI improved practice, 2) clinicians with additional training or experience with mental illness or CPI who spend more time in consultation, and 3) positive beliefs and attitudes towards PWSMI & CPI improved collaboration between clinical teams.

*Positive beliefs and attitudes towards PWSMI & CPI improved collaboration between clinical teams*. Clinicians with positive beliefs and attitudes towards PWSMI & CPI were more likely to report good relationships and higher levels of collaboration between PCPs and the mental health team. This improved healthcare access for patients on the dimensions of approachability and appropriateness of the health service. Some health professionals expressed that when communication channels were open and dialogue was ongoing between PCPs and the mental health team, patients received better access and quality of care because there was a sense of reciprocity between teams and a better platform for patient advocacy: *“I think when you have a good rapport with your colleagues…they send their patients and you be an ally for them [their patients]*, *then they would be more willing to see your patients and care for them also*.*” (Psych #7)*.

**Fig 2:**
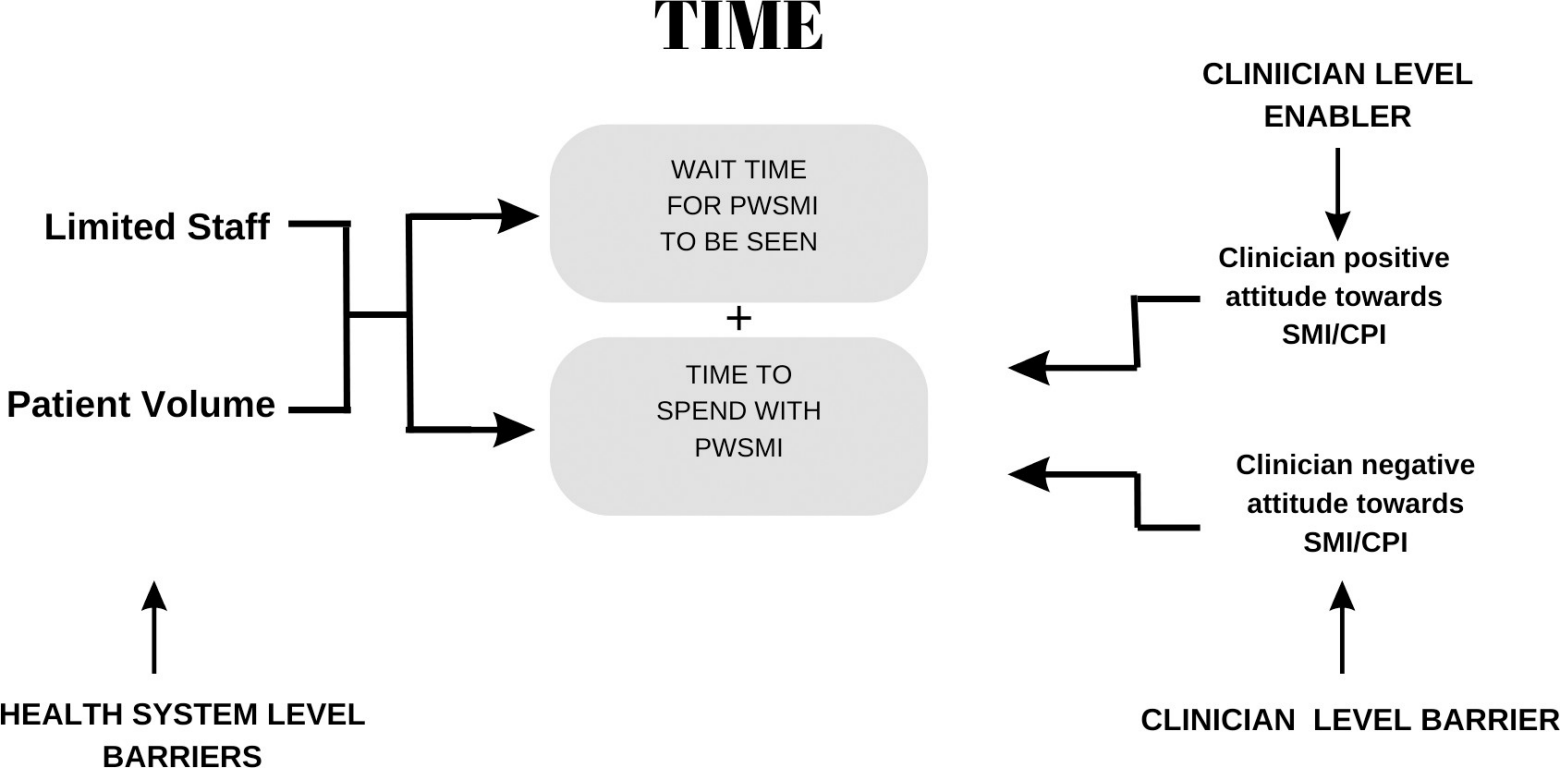
Time as an enabler and barrier to healthcare access across the levels of the health system and clinician.

*Clinicians with additional training or experience with mental illness/CPI who spend more time in consultation*. Time was previously noted as a major barrier however, the barrier of time was reduced among PCPs with additional training in mental health or previous experience working directly with the mental health team (Fig 2). In their practice, these PCPs reported screening and initiating treatment for mental illness, spending more time with PWSMI and following up on both the mental and physical health of patients. When giving referrals to the mental health service, these PCPs spent time explaining the reason for referral and encouraging patients to attend clinic appointments: *“I feel that every interview I should be able to add value to patients and can add a lot of value from counselling a lot of social barriers does exist so I tend to ask about what’s happening*.*” (PCP #8)*. Similarly, Psychs who had previous experience working in physical health care before pursuing Psychiatry reported spending more time enquiring about and investigating the physical health concerns of their patients with PWSMI.

*Positive beliefs and attitudes towards PWSMI & CPI improved practice*. Primary care physicians who expressed positive beliefs and attitudes towards the treatment of PWSMI indicated a greater understanding of mental illness, the bidirectional nature of mental illness and CPIs, and expressed greater investment in rapport with patients. Likewise, all mental health nurses (MHN) believed that monitoring physical health in PWSMI was a part of their role. Psychiatrists who reported being comfortable with managing physical illnesses stated that the physical health of patients was a part of their role and responsibility. They expressed that taking a holistic approach offered greater benefits to patients but required more time, effort, and vigilance (Table 5).

**Table 5:** Illustrative quotes from the SEM level clinician.

Level of socio-ecological model	Themes	Sub-themes	Number of Participants	Illustrative Quote
Clinicians	Clinician related factors that enable healthcare access		28	
		Positive beliefs and attitudes towards patients improved practice	24	It does require more effort and vigilance. It’s positive because if you are effective in treating the whole person then that client has more of a trust in your ability to treat their mental illness as well. The family will have more trust in your ability to be clearly on the client side for their well-being. So they will see the care and consideration put forth, that maybe, is perhaps a breath of fresh air, when they compare it to other experiences they’ve had where they just feel rushed along and passed on. (Psychs #2)
		Clinicians with additional training/experience spend more time	14	In-text
		Positive beliefs and attitudes towards patients improved collaboration	10	In-text
Clinicians	Clinician related factors that act as barriers to healthcare access		25	
		Clinician’s negative beliefs and attitudes towards SMI or CPI management negatively affected practice	22	I think there’s a lot of discomfort or let me call it discomfort amongst all our primary care physicians around mental health in general and it’s not something that persons…..They’re not comfortable with it and as such they either don’t screen for it or they don’t recognize that this is why the patient is/maybe not compliant. (HPM #1)
		Lack of additional training and experience in MI or CPI negatively affected practice	13	…patient would knock on your door. You’re not a psychiatrist and based on their interactions you would pick up that there’s something mentally wrong with them but as far as you’re concerned your hands off because you don’t have any mental health training and you don’t really make an appropriate referral and as a result it does a disservice to both you and the patients. (PCP # 9)

#### Clinician related factors that act as barriers to healthcare access

Two clinician related factors were discussed as barriers to healthcare access for PWSMI & CPI: (1) Clinician negative beliefs, and attitudes towards SMI or CPI management negatively affected practice, and (2) lack of additional training and experience working in mental illness or CPI negatively affected practice.

*Clinician’s negative beliefs and attitudes towards SMI or CPI management negatively affected practice*. Health professionals indicated that negative beliefs and attitudes towards SMI were pervasive in the public health sector (Table 5). Among PCPs, those who believed PWSMI were ‘unpredictable’ expressed ‘discomfort’ treating them unless they were ‘stable’. These PCPs tended to immediately refer patients with suspected mental illnesses, often without conducting detailed screening and medical investigations. Likewise, Psychs who believed that physical health care was outside their scope stated that they would not routinely screen or monitor physical health problems or CPIs and would refer patients who presented with those concerns to PCPs or other specialists.

Clinicians in this category also indicated poorer communication and collaboration between PCPs and mental health teams with each team treating PWSMI & CPI separately. This approach negatively affected PWSMI & CPI access to appropriate healthcare that would facilitate early detection and treatment of developing mental or physical illnesses.

*Lack of additional training and experience in MI or CPI negatively affected practice*. Accessibility to appropriate healthcare was negatively affected by PCPs who indicated their reluctance to undergo screening and initiate management of mental illness due to a lack of knowledge, confidence, and understanding about mental illness (Table 5). Likewise, Psychs who were reluctant to undergo screening and initiate management of CPIs associated this with a lack of current knowledge on the procedures and treatments for CPIs: *“I don’t feel kind of very comfortable in starting the process*. *I will*, *but I don’t feel like I’m up to date in the different criteria” (Psych #7)*.

### Family & community

At the level of family and community, access to healthcare was affected by the amount of social support available to PWSMI & CPI. Social support existed in the form of family, friends, church members, neighbours, and community members. The presence or absence of social support was related to the individual abilities aspect of healthcare access.

#### The presence of social support as an enabler of healthcare access

The presence of social support enabled healthcare access for PWSMI & CPI. Social support was explained in the form of financial, physical, emotional, and/or spiritual support. Financial support included money for transportation for appointments and to purchase medication and food. Physical support consisted of accompanying PWSMI to the clinic, advocating for care, and aiding with treatment compliance. Emotional support involved check-ins, listening to concerns, providing encouragement, and demonstrating concern for their well-being. Spiritual support related to facilitating PWSMI & CPI’s church attendance and praying for their wellness (Table 6).

**Table 6:** Illustrative quotes from the SEM level of family & community.

Themes	Sub-themes	Number of Participants	Illustrative Quote
***The presence of social support as an enabler of healthcare access***		32	I have a friend of mine who is like a prayer partner that we pray every Sunday over the phone and they pray for my needs and I pray for their needs so there is moral support there. Financial support, there are some friends that have been very helpful over the years and they continue to help me… (PWSMI #2).
The absence of social support as a barrier to healthcare access		27	The elderly are coming to the Health Centre alone so that is something that does affect care because if a diabetic who is on insulin [is] not taking their correct insulin because they can’t see properly they don’t have anybody to give it to them- that’s an issue. That affects care for that patient, so a lack of a support system and also resources, if you don’t have resources that always challenges the care that you can get the patients. (PCP #8)

**Note:** dominant enablers and barriers are written in bold italics.

Among PWSMI & CPI, those who had social support whether family, friends, or community support also stated that managing their comorbidities was ‘easy’ and they experienced better healthcare access. Social support helped to address aspects of all five individual abilities that contribute to healthcare access as it allowed PWSMI & CPI to be able to perceive, seek, reach, pay, and engage with health services.

#### The absence of social support as a barrier to healthcare access

When social support was absent, this presented a dominant barrier to healthcare access for PWSMI & CPI. Those who reported limited or no social support also reported poorer health and expressed that it was ‘difficult’ to manage their conditions. The lack of social support reduced PWSMI & CPI’s ability to perceive, seek, reach, pay, and engage with healthcare. This was true among the elderly and those who lived alone, lived far away from family, or did not have family or other social support (Table 6).

### Individual

At the individual level, several individual characteristics act as enablers or barriers to PWSMI & CPI’s ability to perceive, seek, reach, pay, and engage with healthcare.

#### Individual characteristics that enable healthcare access

The individual characteristics that facilitated healthcare access for PWSMI & CPI included: (1) being female and/or having completed higher education levels, (2) positive beliefs, attitudes, and experiences with health services, (3) adequate financial capacity and (4) possessing adequate self-management skills.

*Being female and/or having higher education*. Across participant groups, it was noted that females and people with higher levels of educational attainment experienced better healthcare access. Participants attributed this to having health literacy and the willingness to seek help (Table 7).

**Table 7:** Illustrative quotes from the SEM level of the individual.

Level of socio-ecological model	Themes	Sub-themes	Number of Participants	Illustrative Quote
Individual	Individual characteristics that enable healthcare access		23	
		Being female and/or having completed higher education levels	17	Well [regarding] the educational level, those that are more educated most times I think those persons with tertiary education, tend to be more aware of it [CPI]. I think those who have a higher education level, tend to be more vocal as it relates to their underlying illness. So maybe I don’t know if it’s because they are talking more about it, why it was picked up more… as opposed to seeing someone who is not as educated, somewhat illiterate, but just know that they have a mental illness and just coming in for that. (Psych # 6)
		Positive beliefs, attitudes, and experiences with health services	12	“Whenever I have the [clinic appointment] date and I have to be at work… I would explain it and they would look after me and the nurse there, she would call and get my medication for me and bring them for me because I work a little way from her [where she lives]. (PWSMI #14)
		Adequate financial capacity	10	I don’t really have to depend on somebody and because I work, I am not so stressed to manage the diabetes. So, it really doesn’t affect me very much because as long as I am on my medication, I am okay. (PWSMI # 23)
		Possessing adequate self-management skills	8	In-text
	Individual characteristics that act as barriers to healthcare access		51	
		***Poverty***	48	***Clinician*:** If you’re working in a public system, you know, the type of persons you’re going to encounter are from less fortunate type of social economic background and possibly doesn’t have the same type of financial resources to allocate to private care. (Psych #5).
				***Patient*:** It’s difficult, because sometimes I have to buy the medications and… [my] husband not working… and I just doing a part time job, with the [government insurance card] NHF card I only have to pay $200 [$1.30 USD] for it but sometimes I don’t have the money. (PWSMI #4)
		Being male and /or lower education	11	In-text
		Negative, beliefs and attitudes towards and experiences with health services	9	The issues are if you have a mental illness and a physical illness depending on how acute the physical illness is, you’re going to suffer. If you have a mental illness, you are not an easy patient, you’re going to give trouble and people don’t want trouble. People get very impatient with you, as a mentally ill person who has a physical illness. (Caregiver #1).

**Note**: dominant enablers and barriers are written in bold italics.

*Positive beliefs*, *attitudes*, *and experiences with health services*. PWSMI & CPI and caregivers who had positive sentiments about the public health system tended to report greater healthcare access. The relationship between PWSMI & CPI and clinicians was also important to healthcare access, with good relationships facilitating trust and greater treatment adherence. This worked especially well in settings where clinicians were familiar with patients and their circumstances and took steps to accommodate them (Table 7). These PWSMI & CPI expressed gratitude for free healthcare services and most reported easy engagement with healthcare services.

*Adequate financial capacity*. Among PWSMI & CPI, those who had financial resources whether through employment or social support were able to reach and pay for costs of transportation to attend their appointments, purchase food and medication as needed, and provide for their basic needs (Table 7).

*Possessing adequate self-management skills*. A few participants in the PWSM & CPI indicated that they self-managed and independently followed through on treatment directives. *“I am very much able to monitor myself*, *I can do it myself*.*” (PWSMI #11)*. Self-management skills enhanced their ability to perceive, seek, and engage with healthcare, thus improving their healthcare access. The ability to self-manage was complemented by a belief in the benefits of medication for treating their comorbidities. For others, self-management was aided by their ‘faith’ in God. Other PWSMI & CPI indicated that social support enabled their self-management activities.

#### Individual characteristics that act as barriers to healthcare access

Individual factors that inhibit healthcare access included: (1) poverty; (2) Being male and/or having lower educational attainment, and (3) negative, beliefs, attitudes, and experiences with health services.

*Poverty*. Across all participant groups, poverty was recognised as the main barrier to healthcare access for PWSMI & CPI. Most PWSMI & CPI in the study indicated that they were unemployed and lacked adequate financial resources to meet their needs. Health professionals indicated public health services are primarily accessed by people from lower socio-economic status because they cannot afford private healthcare which is usually considered as preferred to public healthcare (Table 7).

Participants indicated that poverty affected healthcare access directly and indirectly. Directly and as previously noted, PWSMI & CPI who lacked financial resources, were often unable to pay the costs of transportation or food for the day at the clinic. In addition, because physical and mental health clinics often took place on different days in the community health centres, patients could not afford to attend both clinics and would select one, but not both. If medication was not freely available, many PWSMI went without. This was true even for those with government insurance cards who had to pay very little for the medication (Table 7).

The experience of poverty resulted in some PWSMI & CPI being homelessness, having unstable residences, or having a home but living in poverty. They could not afford to adhere to medical advice related to nutrition and self-care because they could not afford healthy foods and other necessities. Consequently, poverty indirectly affected healthcare access as it obstructed PWSMI & CPI’s ability to reach, pay, and engage with healthcare services. While a few PWSMI & CPI were able to access government welfare assistance programs to help with their basic needs, others were unsure of how to access help. This situation was exacerbated by the COVID-19 pandemic, which led to some PWSMI & CPI and/or their relatives who supported them losing their jobs, driving some patients into greater poverty.

*Being male and /or having lower educational attainment*. Across clinician groups, PWSMI & CPI who were males and those had limited education, including not completing school, were seen to have lower health literacy and awareness, and be less likely to perceive healthcare needs, seek help, and engage with health services before symptoms were uncontrolled:*“…men in Jamaica have poor health seeking behaviour so majority of the clinics are dominated by women” (PCP #4)*.

*Negative*, *beliefs and attitudes towards and experiences with health services*. A few of the PWSMI & CPI and caregivers expressed being mistrustful of the health system. They felt that clinicians were hurried or disinterested in them and or did not feel their prescribed psychiatric medication was helpful. This negatively affected their ability to engage with health services. Others reported negative experiences with clinicians and administrative staff in the health system, marked by a lack of competency, empathy, and patience (Table 7).

## Discussion

The findings of this study identify five levels of a socio-ecological model that included enablers and/or barriers to public healthcare access for PWSMI & CPI in Jamaica. The primary enabler of access reported across all participant groups was the free public healthcare system as it provided a policy framework for physical and mental healthcare, community-based co-located mental and physical primary healthcare services, community outreach for PWSMI & CPI, and no-cost medication. In this way, it helped to address some issues of healthcare approachability, availability and accommodation, affordability and appropriateness of health services as it allowed PWSMI & CPI to obtain consultancy and treatment for their health concerns close to their homes [20, 22].

Despite the benefits of a free healthcare system, poverty, followed by stigma and discrimination were identified as the most pronounced barriers to healthcare access for PWSMI & CPI. This finding reflects other international studies, which highlight the commonplace nature of poverty among PWSMI [70, 71]. Poverty and mental illness have been found to have a bidirectional relationship [41]. People who grow up in poverty are more prone to develop mental illness due to poor environmental conditions [41, 72]. On the other hand, people from middle or upper-income brackets who develop mental illness are likely to descend into poverty due to changes in the capacity to work as well as due to the effects stigma, discrimination, social isolation and exclusion [41, 72]. Although poverty was presented as an individual characteristic in the SEM, it is understood that poverty is a complex and multidimensional social issue that is also associated with stigma and discrimination [70]. For instance, our findings indicated that most PWSMI &CPI had lower education levels and were unemployed and reported having no sustainable means to take care of themselves. Thus, although healthcare services were free and deemed affordable, they still were not consistently accessible to the poorest of the poor in this patient population. Additionally, lower educational attainment is common among people who experience poverty and has been linked to lower levels of health literacy and negative health beliefs and attitudes towards healthcare resulting in delayed utilisation of health services [73].

However, the availability of social support was found to improve healthcare access for PWSMI & CPI in this study. Positive participation of social support improves healthcare access for PWSMI &CPI [26, 74]. In the context of low material resources, people may tap into social support as a means of accessing needed resources [26]. Yet, the benefits of social support extended beyond financial resources in this study and were physical, emotional, and spiritual and aided individuals’ ability to access healthcare in various ways.

In contrast, a connection was found between PWSMI & CPI having a lack of financial means, perceiving their comorbidities as difficult to manage, feeling isolated and the absence of social support in this study. Other literature has identified this pattern and has demonstrated a connection between poverty, social exclusion, and isolation experienced by PWSMI to stigma and discrimination [70, 75]. Our findings indicated that stigma and discrimination were reported to exist across all levels of the socio-ecological system represented as interpersonal, structural, and intrapersonal stigma. Stigma and discrimination toward PWSMI have been described as enforcing and re-enforcing marginalisation and social exclusion [33, 70]. Thus serving to perpetuate health inequalities for PWSMI and result in delayed utilisation of health services, diagnostic overshadowing, poorer screening and management of health concerns which leads to underdiagnoses of CPIs and poorer health outcomes [76, 77]. These findings suggest the need for the development of strategies that can address issues of poverty that interplay with stigma and discrimination to directly and indirectly hinder healthcare access for this vulnerable population.

Some health system factors further contributed to reducing healthcare access for PWSMI &CPI. For instance, the emphasis on the bio-medical approach was seen as a barrier to the accessibility of appropriate healthcare. The bio-medical approach has been criticised for its lack of recognition and attention to social and environmental factors in creating, maintaining and worsening the health care access and negative health outcomes of PWSMI & CPI [70]. The application of a bio-medical approach to a complex socio-ecological system serves to affirm a health system focus on individual symptom reduction and medical maintenance [70]. This is central finding of the study considering that poverty, stigma and discrimination and a lack of social support were the primary barriers to healthcare access, which are all social determinants of health recognised to perpetuate health inequalities among PWSMI & CPI [41].

Furthermore, the bio-medical approach relies predominantly on physicians and nurses to provide health services. Participants indicated that limited staff and high patient volume extended the wait time for patients to be seen and reduced the time clinicians spent in consultation. This can present substantial limitations to health service delivery especially in the context of limited resources [78]. It also has clear implications for screening, early detection and treatment of patients’ health concerns which is especially important when managing patients with comorbidities or complex disabilities [79]. However, clinicians who had more training or experience with mental illness/CPI spent more time with patients and were considered to have better attitudes and practices related to the care of PWSMI & CPI. When clinicians display interest in holistic care, demonstrate competency around mental health and physical health and engage in screening and management procedures, access and outcomes are improved for PWSMI [27, 28, 42]. These findings highlight the importance of additional training and experience in reducing stigma and discrimination and improving the competencies and practices of clinicians working with this patient population and enhancing healthcare access for PWSMI & CPI [80–82].

Nevertheless, the shortfalls of the bio-medical approach were still pronounced as clinicians who expressed confidence and competence in managing PWSMI & CPI were in the minority and even then, they acknowledged their limitations in addressing the social and environmental needs of patients. Additionally, the bio-medical approach has been viewed as fostering fragmentation of health through over specialisation, over reliance on medication as treatment, a lack of holistic care, ongoing paternalistic relationships between doctors and patients that disempower individuals and reduce their right and opportunity for self-direction and self-advocacy around their healthcare preferences [83, 84].

Contemporary approaches to mental healthcare such as the social model of health and recovery-oriented approaches may improve healthcare access and outcomes for PWSMI & CPI [53, 85, 86]. These approaches support the inclusion of multidisciplinary teams; something highlighted as improving healthcare access by some participants in this study. Indeed, best practice guidelines for the treatment of PWSMI & CPI recommend the involvement of multidisciplinary teams [19]. These contemporary approaches also reduce the burden of care for medical practitioners, help to address social aspects of healthcare, emphasising the empowerment of PWSMI & CPI towards self-management through psycho-education, and skill building to experience better health and quality of life [86–88].

The findings of this study suggest the need for a reconceptualisation of healthcare access for PWSMI & CPI in Jamaica to build on areas of strength and to develop strategies that address the barriers that exist across the SEM. The challenges that affect healthcare access for PWSMI & CPI are extend beyond the health sector and require the involvement of multi-sectoral stakeholders to ensure strategies are impactful and sustainable [89]. Within health service delivery, there is a need for a graduation from co-location of services towards further integration of mental health care with the inclusion of more multidisciplinary teams (with social workers, psychologists and occupational therapies), better co-ordination and collaboration of clinicians and person-centred care to meet the healthcare access needs of PWSMI & CPI [90].

### Strengths & limitations

A constructivist grounded theory approach was utilised in this study to allow for a comprehensive exploration of enablers and barriers to public healthcare access for PWSMI & CPI. Purposive sampling was used to identify key participant groups that could provide meaningful insight into the research area [91]. The findings of the study are situated in the context of the time, place and culture of Jamaica and were representative of this setting [55]. However, only the perspectives of selected health professionals were considered in the study, inclusion of other health professionals such as nurses, and administrative staff would have enhanced these findings. Additionally, interviews were conducted via telephone or video conferences and therefore people without access to this technology were not able to take part in the study.

### Conclusion

The enablers and barriers to healthcare access for PWSMI & CPI have been found to exist across a five-level SEM. The provision of free public healthcare and primary healthcare through community-based co-located mental and physical health services represented the main enabler to healthcare access for this population. However, issues of poverty, stigma, and discrimination were identified as formidable barriers to healthcare access and reduced the effectual gains made by other enablers. Given the socio-cultural basis, the current bio-medical approach to healthcare was deemed inadequate to help PWSMI &CPI overcome the barriers they experience to healthcare access particularly in the realm of the social determinants of health. There is a clear need for the identification of strategies that address the barriers to healthcare access across the SEM, this would likely include a reconceptualisation of the current healthcare approach as well as incorporate multi-sectoral approaches that span beyond the health sector. There is also a need for further research that expands beyond healthcare access for PWSMI & CPI to exploring quality of life and health outcomes for this vulnerable population.

## Supporting information

S1 Questionnaire (DOCX)
